# Tool Wear Monitoring in Milling Based on Fine-Grained Image Classification of Machined Surface Images

**DOI:** 10.3390/s22218416

**Published:** 2022-11-02

**Authors:** Jing Yang, Jian Duan, Tianxiang Li, Cheng Hu, Jianqiang Liang, Tielin Shi

**Affiliations:** School of Mechanical Science and Engineering, Huazhong University of Science and Technology, 1037 Luoyu Road, Wuhan 430074, China

**Keywords:** tool wear, tool condition monitoring, machined surface images, fine-grained image classification, channel attention, convolutional neural network (CNN)

## Abstract

Cutting tool wear state assessment during the manufacturing process is extremely significant. The primary purpose of this study is to monitor tool wear to ensure timely tool change and avoid excessive tool wear or sudden tool breakage, which causes workpiece waste and could even damage the machine. Therefore, an intelligent system, that is efficient and precise, needs to be designed for addressing these problems. In our study, an end-to-end improved fine-grained image classification method is employed for workpiece surface-based tool wear monitoring, which is named efficient channel attention destruction and construction learning (ECADCL). The proposed method uses a feature extraction module to extract features from the input image and its corrupted images, and adversarial learning is used to avoid learning noise from corrupted images while extracting semantic features by reconstructing the corrupted images. Finally, a decision module predicts the label based on the learned features. Moreover, the feature extraction module combines a local cross-channel interaction attention mechanism without dimensionality reduction to characterize representative information. A milling dataset is conducted based on the machined surface images for monitoring tool wear conditions. The experimental results indicated that the proposed system can effectively assess the wear state of the tool.

## 1. Introduction

The rapid development of advanced manufacturing is closely related to a country’s economy [1]. Milling is an indispensable part of modern manufacturing industries and optimizing its machining process is crucial to achieving substantial economic benefits and improving product quality. Manufacturing cost is mainly affected by the cutting tool, power, machining efficiency, and machined surface quality [2]. Cutting tool wear condition is an important factor during the manufacturing process, which directly affects the quality of products and the operation of equipment. Moreover, monitoring tool wear condition is necessary to ensure timely tool change; an early tool replacement increases cost, while a tool replaced too late causes reduced workpiece quality and even damages the machine tool. In the machining process, the total processing cost of cutting tools and replacing them ranges from 3% to 12% [3]; at the same time, the downtime caused by cutting tools can represent up to 20% of total machining downtime [4]. Tool wear is a normal phenomenon because the tool is in contact with the chip and workpieces. Moreover, the tools undergoes various failures mechanisms, such as adhesion, abrasion, chipping, diffusion, and plastic deformation. Due to the highly complex physics behind tool wear, it is challenging to avoid tool wear. Therefore, tool wear monitoring is essential in advanced manufacturing to optimize the machining process and maintain the quality of the manufactured product. For these purposes, it is crucial to develop an automated and accurate tool wear monitoring system to generate warnings for tool wear, which has been the focus of various research studies in tool wear monitoring.

Recently, cutting tool condition monitoring has focused chiefly on extracting degradation information by various physical properties, and the research methodologies have been divided into the following categories [5]: direct approaches, in which a machine vision system as a common method [6,7] is used to directly measure the areas of tool wear for evaluating tool conditions; and indirect approaches, in which the wear state is evaluated by analyzing signals from various sensors, such as machining force [8], vibration [9], temperature [10], acoustic emissions (AE) [11], and machined surface roughness [12,13,14,15,16,17]. Surface texture images, as relatively easily obtained monitoring data, can be extracted by cost-effective data acquisition devices to provide rich information for diagnosis and monitoring applications. The machined surfaces’ texture not only directly realizes the evaluation of machined surface quality but is also indirectly utilized to reflect various types of tool wear, such as flank wear, crater wear, nose wear, fracture, and breakage. Compared with the abovementioned methods, the most notable advantages of analyzing machined surface images for monitoring tool wear states are non-invasive, low-cost, and flexible technique.

The machined surface images could provide rich geometrical characteristics for diagnosing the soundness of the state of cutting tools [18]. The massive and unstructured raw image data brings new opportunities and challenges to vison-based tool condition monitoring. Because machine vision-based tool condition monitor methods can learn the texture characteristics of an image from massive image data and automatically build the corresponding monitoring models, these have attracted increasing attention in recent vision-based tool condition monitoring studies. The machine vision approach relies on handcrafted feature design to extract sensitive degradation features using prior knowledge and expertise from the acquired monitoring data. Then, the extracted features are sent into machine learning models to assess the target values. At present, there is a rich research literature on the topic of machine learning surface texture inspection for tool wear assessment. Bhat et al. [12] presented a support vector machine (SVM) model to predict the state of tools using features extracted from the grey level co-occurrence matrix (GLCM) of machined surface images. Dutta et al. [13,14] proposed a support vector machine-based regression model to assess tool flank wear using extracted features from turned surface images. Kassim et al. [15] developed a run-length statistical method to monitor the tool condition based on the machined surface images by using a machined vision technique. Riego et al. [16] designed an extremely randomized tree algorithm to monitor the wear state by means of supervised classification. Li et al. [17] studied a micro-vision system to monitor the wear of a cutting tool, which combines an insert image with workpiece texture. These methods aim to develop an intelligent method to monitor the level of tool wear through the machined surface. Although these automated monitoring systems employed handcrafted feature extraction by machine vision to achieve decent results for monitoring tool wear, which requires significant computational effort and related knowledge, they are less intelligent.

To help enhance the intelligence capability of tool monitoring systems, a new branch of artificial intelligence called deep learning [19] has widely emerged in tool condition detection fields and has provided excellent results. Deep learning is a particular kind of machine learning structure with more powerful feature learning abilities, including deep belief networks (DBNs), recurrent neural networks (RNNs), and convolutional neural network (CNNs). It achieves advanced accuracy in various tasks, such as computer vision [20], speech recognition [21], and natural language processing [22]. Compared with traditional handcrafted feature extraction, deep learning-based tool wear monitoring studies [23,24,25,26,27,28] realized the complex correlation between the input (signal data) and the target output values (tool wear). These papers observed that the latent features are automatically learned from raw data for deep learning technology, which is advantageous over manually designed features. Although deep learning-based tool condition monitoring has been developed in recent years, very few studies have focused on deep learning-based machined surface monitoring methods. In 2021, Kumar et al. [29] proposed a deep CNN architecture for intelligent wear monitoring of a cutting tool using machined surface images in turning. The model applied preprocessing images as an input to overcome inhomogeneous equalization. Although the model has achieved promising monitoring results, the preprocessing operation is not intelligent enough. In this paper, an end-to-end intelligence method should be proposed for tool condition monitoring, which creates direct mapping from raw data (machined surface images) to the needed results (the level of cutting wear). The workpiece textures with different tool wear states are characterized by visually shared global texture structure. The wear category is to be recognized by local regions with smaller visual differences. Fine-grained image classification, as a challenging task in computer vision, can distinguish different local details under the same global structure. The discriminative local regions play a critical factor in fine-grained classification and are identified to classify the target categories. Therefore, fine-grained classification has been introduced to recognize similar workpiece textures to identify the tool wear state.

The fine-grained image classification method has been widely applied in various classification tasks with the same global structure (such as animals, product brands, and vehicles) [30,31,32,33,34,35]. Deconstruction and construction learning (DCL) [36] is a newly proposed fine-grained classification model and differs from traditional classification methods with significant differences between categories, which aim to extract discriminative features from similar global structures. The architecture for DCL includes both destruction and construction streams, where the former is used to enhance recognition robustness while the latter is used to simulate the semantic correlations between image regions. As a result, DCL, as an end-to-end approach, may be readily learned without manual intervention. It, in particular, is light in weight, fast in reasoning, and practical. In this study, an intelligent method was proposed focusing on cutting tool wear monitoring from machined surface images based on the architecture of a CNN with fine-grained classification.

An attention mechanism is used to ensure that most salient information is noticed, which has been proven effective in many previous studies [37,38,39]. Hu et al. [40] developed the squeeze-and-excitation (SE) module to learn channel features by using interchannel relationships. However, describing the learned features by dimensionality reduction is suboptimal. Wang et al. [41] presented an efficient channel attention (ECA) module as an improvement of the squeeze-and-excitation module.

In this article, efficient channel attention destruction-construction learning (ECADCL) is proposed to monitor the tool wear state (sharp, normal, or dull) from machined surface images. The proposed ECADCL, including the feature extraction module, destruction-construction module, and decision module, combines the conventional CNN and the idea of DCL for identifying the local detail from a similar global structure. Considering the ability to learn feature representation, the ECA module is introduced into the feature extraction module and named ECACNN. Ultimately, the experimental results confirm that ECADCL offers better tool wear monitoring performance than the existing methods.

The main contents of this study are organized as follows.

(1) The architecture of ECACNN is structured by combining a typical CNN and attention mechanisms. This architecture can enhance the channel relationships of features and extract efficient information from texture images.
(2) Based on fine-grained image recognition, ECADCL is designed to enhance the representation of local details.
(3) An automatic monitoring system, which is a combination of the designed algorithms and the image acquisition system, is designed to detect the tool conditions based on machined surface images.
(4) The experiments verify that the proposed method is accurate and effective. Compared with handcrafted feature extraction methods and conventional CNN methods, we show that the proposed ECADCL can obtain competitive performance with handcrafted feature extraction and solve local feature recognition problems compared to conventional CNNs.

The rest of this article is organized as follows. In Section 2, the experimental system is described. In Section 3, the proposed ECADCL technique is elaborated. Section 4 presents the experimental results. Section 5 concludes the article.

## 2. System Setup

The experimental data used in this article are from a TC500 high-speed CNC milling machine, which uses uncoated carbide end mill inserts (ZMCC PML-4E-D6) with a four-insert-type end mill cutter 6 mm in diameter. The work material used when collecting the data was SUS 316 steel. The parameter combinations for the conducted experiments are listed in Table 1.

**Figure 1:**
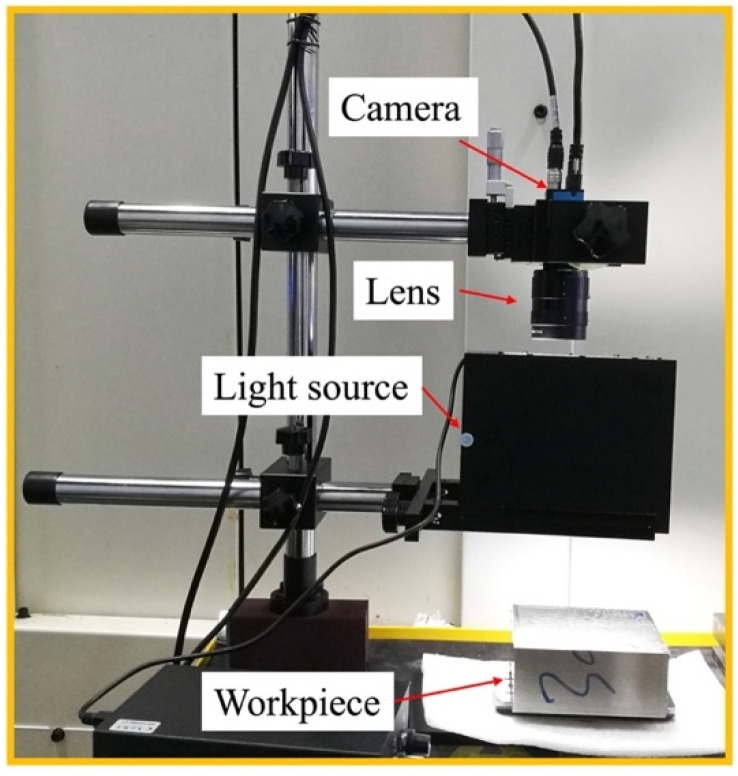
Prototype of the machined surface image acquisition system.

In the experiments, with the goal of minimizing the complexity of designing computer vision algorithms, using a camera precisely acquires a high-quality image of the workpiece surface. We created an image acquisition system that included a camera and an illuminator to capture images of all machined surfaces. Figure 1 illustrates that a complementary metal-oxide-semiconductor (CMOS) industrial camera (DMK 33GX183), a coaxial light source, and a workpiece are the key components of the image acquisition system. The CMOS camera and coaxial light source were mounted in a vertical manner, and the relative distance between the workpiece and camera was captured at certain predefined positions. The surfaces of the resulting machined workpiece were collected as 5472 × 3648-pixel red-green-blue (RGB) digital images by the camera, which was connected to a computer equipped with image acquisition capabilities, at five different positions relative to the machined surface.

**Figure 2:**
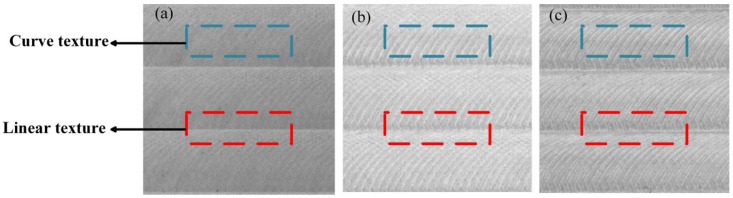
Image samples corresponding to different tool wear states: (**a**) sharp; (**b**) normal; and (**c**) dull.

In our study, we used the average tool flank wear (${\mathrm{VB}}_{\mathrm{average}}$) as the measurement standard, while the value of ${\mathrm{VB}}_{\mathrm{average}}$ was measured using a Keyence VHX-700F microscope. Flank wear appears to be caused by the wear of the cutter edge, which will affect the surface texture wear value. Thus, according to the tool wear value, the evaluation criteria of tool condition can be divided into three categories: sharp (${\mathrm{VB}}_{\mathrm{average}}$ < 50 μm), regular (50 μm <${\;\mathrm{VB}}_{\mathrm{average}}$ < 80 μm), and dull (${\mathrm{VB}}_{\mathrm{average}}$ > 80 μm), which are shown in Figure 2. The surface image textures are divided into fine-grained curve texture and coarse-grained linear texture.

## 3. Methodology

**Figure 3:**
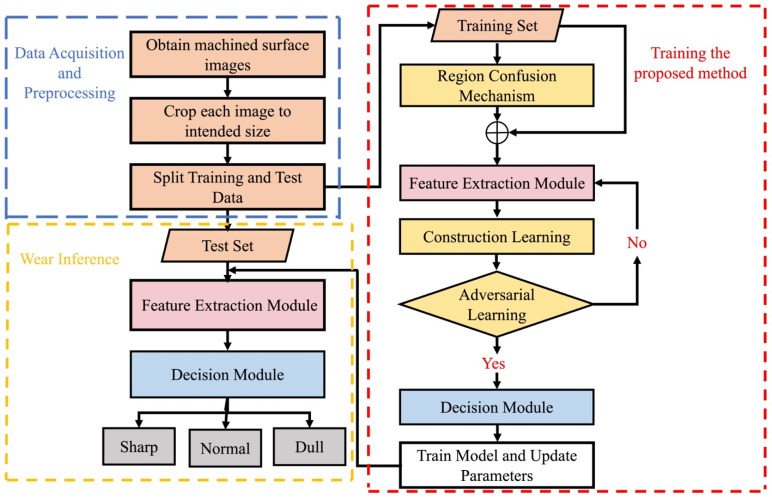
Flow chart of the proposed method for tool wear monitoring.

In this work, cutting wear monitoring based on the machined surface is regarded as an image classification problem. We aim to design an end-to-end intelligent model for tool wear monitoring that directly maps the input data to the target output. The model is represented as C(X, Y | θ), where X represents a set of input images, Y denotes the label of X, and θ denotes all the learnable parameters. It should be noted that the different levels of wear on machined surface images have the same global structure and differ only in specific local details. In order to find the discriminating area among different tool wear states, the fined-grained method is introduced for realizing the monitoring tool condition. The procedure of this method is shown in Figure 3. Basically, it includes three stages: acquisition, training, and inference. The first part consists of data acquisition and data processing. In the second part, the proposed method applied the channel attention mechanism and the destruction-construction structure, and the training process mainly consists of feature extraction with attention mechanism, destruction learning (DL), and construction learning (CL). In the destruction process, the region confusion mechanism (RCM) disrupted the global structure into the local regions and finds the discrimination area by the feature extraction module. Nevertheless, adversarial learning is introduced into the DL part, which prevents the noise emerged by overfitting RCM from negatively affecting network learning. In addition, CL induces the feature extraction module to learn the semantic correlative among local regions to reconstitute the original image. In the inference process, test samples have been sent into the trained feature extraction network and obtained the classification results.

**Figure 4:**
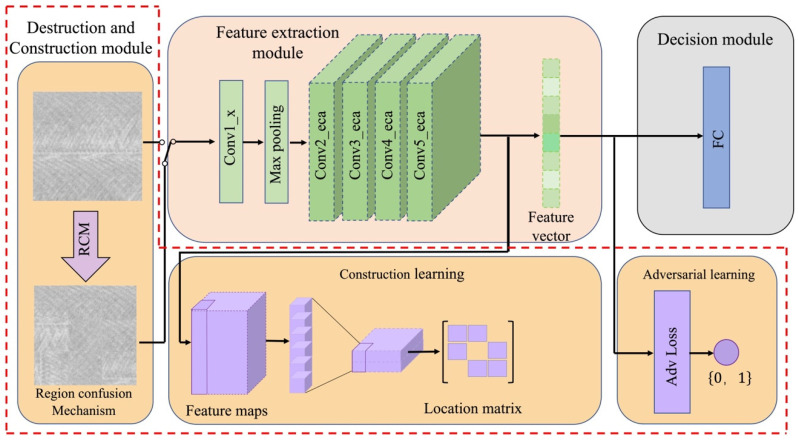
The architecture of ECADCL, which includes three modules: (1) feature extraction module; (2) destruction and construction module; (3) Decision module.

In this paper, ECADCL is designed to learn the local feature space based on the disruption of the global structure. The overall architecture structure of ECADCL is illustrated in Figure 4, and in the following subsections, we will present our proposed tool wear monitoring method based on machined surface images in detail.

### 3.1. Feature Extraction Module

Our suggested framework is a typical CNN, which can be seen in Figure 3. The creation of a network structure is a significant challenge in practical applications. However, transfer learning has been found to be a well-performing way to overcome this issue. In this study, the ECADCL architecture is developed, relying on three types of famous networks in transfer learning, namely, AlexNet [42], VGG-16 [43], and ResNet-18 [44], which exhibit excellent performance for image recognition. Comparing the results of the above three famous networks, the ResNet18 is selected as the basic framework, and the details of the proposed model architecture are listed in Table 2.

As illustrated in Table 2, the feature extraction module architecture, mainly constituted by one input layer and five convolutional blocks, has the same size as the input image, 256×256×3. There is a max pooling layer, 8 ECA layers, and 17 convolutional layers in the five convolutional blocks. Following the first convolutional block, the max pooling layer is responsible for replacing the output for the appropriate places with the overall features of the nearby domains in order to complete the downsampling procedure. In this study, the rectified linear unit (ReLU) activation function is used for nonlinearity. Note that the original image X, its destroyed version ϕ(X), and its ground-truth one-vs.-all labelling Y are combined for training. Therefore, the extracted features may be described by the equation:

(1) $$F_{t}=\langle C_{n}^{m}\left(X\right)C_{n}^{m}(\phi \left(X\right))\rangle =\forall \left(I_{i}{|\theta }_{m}\right)\in {\mathbb{R}}^{W\times H\times N}$$

where $F_{t}$ indicates the learned features, $C_{n}^{m}\left(X\right)$ represents the mth feature of the original image in the nth channels, ∀ denotes the feature extraction procedure, θ represents all the learnable parameters, and the three dimensions indicate the width, height, and number of channels, respectively, of the learned features.

### 3.2. Attention-Based Network

**Figure 5:**
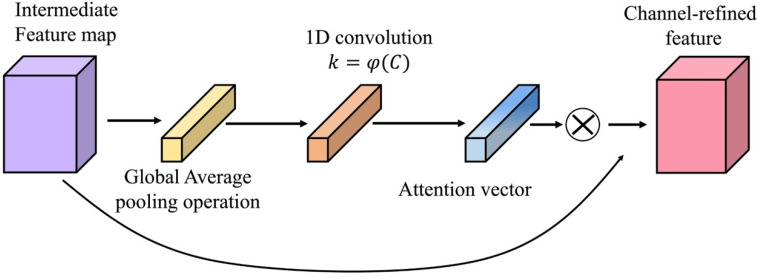
Structure of the ECA module.

The underlying attention mechanisms are inspired by the human visual system, which tends to process the most critical information in a scene rather than attempting to analyze the whole scene simultaneously. Accordingly, an attention mechanism can be introduced into a CNN to concentrate on more representative sections while suppressing less significant information. For this purpose, the ECA module is introduced into our feature extraction module. Referring to Figure 5, EAC is a local cross-channel interaction strategy that can be efficiently implemented without dimensionality reduction to adaptively determine the coverage of local cross-channel interactions.

In the ECA block, the intermediate feature map $F_{t}$ is subjected to the global average pooling (GAP) operation and then generates an aggregated feature vector: $V_{\mathrm{agg}}$. This vector is then input into a fast 1D convolution of size k, which produces the attention vector $A_{\mathrm{eca}}$. Finally, to acquire the channel attention vector $V_{c}$, the attention vector is passed via the sigmoid activation function. Thus, $V_{c}$ can be generated as follows:

(2) $$V_{c}=\sigma \left(C1D_{k}\left(G\left(F_{t}\right)\right)\right)=\sigma \left(C1D_{k}\left(V_{\mathrm{agg}}\right)\right)=\sigma \left(A_{\mathrm{eca}}\right)$$

where G(·) denotes the GAP operator, $C1D_{k}\left(\cdot \right)$ represents a 1D convolutional layer and its kernel size k, and σ(·) is the sigmoid function.

In the ECA block, determining the value of k is another critical issue. Considering a similar principle to group convolutions, different channel dimensions C correspond to different convolution ranges k in feature maps, giving rise to a mapping relationship between k and C. This relationship is characterized by a nonlinear function mapping from kernel size k to channel dimension C, which can be expressed adaptively as follows:

(3) $$k=\varphi \left(C\right)={\left|\frac{\log_{2}\left(C\right)}{\gamma }+\frac{a}{\gamma }\right|}_{\mathrm{odd}}$$

where ${\left|t\right|}_{\mathrm{odd}}$ is the odd integer that is closest to t. As verified in [35], we set γ and a to 2 and 1, respectively. φ(·) represents a nonlinear mapping that relates feature maps with various dimensions of channels and varying interaction ranges, driving the model to adaptively learn the interdependencies between feature channels.

### 3.3. Destruction-Construction Module

As mentioned above, machined surface images are visually similar in terms of their global information. Hence, the proposed algorithm needs to learn to discriminate local details within the same global structure. The destruction-construction strategy is introduced to improve the ECACNN architectures to address this issue. The destruction-construction module consists of three branches: the region confusion mechanism (RCM), adversarial learning, and construction learning. RCM is the key part of the destruction-construction module since it destroys the entire structure by causing localized disruption. To avoid the noise caused by the RCM operation, adversarial learning is recommended during the destruction learning process. Construction learning is proposed for reorganizing previously learned local information in accordance with the semantic relevance among areas. For the input, consisting of the original X, and after RCM obtained destroyed images ϕ(X), they are sent into the feature extraction module to obtain the feature map, which is sent to the construction module, while the adversarial network receives the feature map after average pooling.

For RCM, it is suggested that the spatial distribution of the whole structure be disrupted and allow the local areas to move within a specific range. In the confusion method, any original image X is divided into N × N subregions, each denoted by $X_{i,\;j}$, which corresponds to the horizontal coordinate i and vertical coordinates j. The subregions are rearranged as a destroyed image ϕ(X), which generates a new coordinate:

(4) $$\sigma \left(i,j\right)=\left(\sigma_{j}^{\mathrm{row}}\left(i\right),\sigma_{i}^{\mathrm{col}}\left(j\right)\right)$$

where σ(i,j) represents the subregions at locating (i,j) in the original image X, $\sigma_{j}^{\mathrm{row}}\left(i\right)$ represents a new coordinate $\sigma_{j}^{\mathrm{row}}$ of the areas in the jth row of X by a random vector $q_{\;j}$ of size N, the size of the ith element $q_{\;j,i}=i+r$, with r~U(−k, k) being a uniformly distributed random variable within the stretch of [−k, k], and k as a tunable parameter (1 < k < N) defines the neighborhood range. $\sigma_{i}^{\mathrm{col}}\left(j\right)$ similarly represents a new coordinate $\sigma_{i}^{\mathrm{col}}$ of the area in the ith column of X.

For adversarial learning, a discriminator is designed as a new stream to judge whether the input image X has been destroyed or not. It prevents the RCM-induced noise patterns from shuffling the local regions into the feature map for classification tasks, which can be written as follows:

(5) $$D\left(X,{\;\theta }_{\mathrm{adv}}\right)=\mathrm{softmax}\left(\theta_{\mathrm{adv}}F\left(X,\theta_{i}^{\left[1,m\right]}\right)\right)$$

where $F\left(X,\theta_{i}^{\left[1,m\right]}\right)$ is the feature vector extracted from the outputs of the mth layer in the feature extraction module, $\theta_{i}^{\left[1,m\right]}$ represents the learnable parameters from the 1st layer to the mth layers in the feature extraction module, and $\theta_{\mathrm{adv}}\in {ℛ}^{d\times 2}$ is a linear mapping.

For construction learning, a region alignment network is designed to calculate the positional precision of different regions in the images and simulate the correlations between local regions in an end-to-end fashion. This can be expressed as follows:

(6) $$M\left(X\right)=h\left(F\left(X,\theta_{i}^{\left[1,n\right]}\right),\theta_{\mathrm{loc}}\right)$$

where M(X) represents the two channels of row and column coordinates, h(·) represents the suggested region alignment network, $F\left(X,\theta_{i}^{\left[1,n\right]}\right)$ is the feature vector extracted from the outputs of the nth layer in the feature extraction module, $\theta_{i}^{\left[1,m\right]}$ represents the learnable parameters from the 1st layer to the nth layers in the feature extraction module, and $\theta_{\mathrm{loc}}$ represents the learnable parameters in the region alignment network.

The final decision module, relying on the typical classified design of the network, includes a fully connected layer to predict the output results.

## 4. Case Validation

### 4.1. Dataset

In this study, we acquired a large number of images from the image acquisition system to train the network. Since the quality of the workpiece images is affected by an illuminated environment, the raw images were center-cropped into RGB images with dimensions of 2560 × 2560 pixels to reduce the effect on the brightness uniformity for further analysis. To facilitate the experiment without model overfitting, a cropped RGB image was uniformly cropped to 100 sub-images of 256 × 256 pixels. Eventually, the raw dataset is made from a total of 18,000 images of machined surface images involving four cutting tools. The raw dataset was divided into a training set and a test set, including 12,600 training images and 5400 test images. The distribution of dataset samples is labelled in Table 3.

### 4.2. Implementation Details

The laboratory environment: Python 3.9, PyTorch 1.10.0, and torchvision 0.11.1. For all methods in the experiments, 200 training epochs were performed; the initial learning rate was 0.0001, and it decayed by a factor of 10 at 40, 80, and 120 epochs; and the training set was divided into 64 batches. The number of workers in PyTorch was set to eight for both training and validation. To train the proposed method, in the RCM, the number of regions N must be divisible by the size of an input image, and was set to four in this study. Then, the proposed feature extraction module, ECACNN, was used to extract the feature map from the input, which was sent to the construction module while it was sent to the adversarial network after average pooling. Finally, the feature vector is fed into the decision module to predict the outcome of the classification category. For the testing period, the destruction-construction module is disabled. At the same time, the original images as input are directly sent to the feature extraction module and decision module for the prediction of the results.

### 4.3. Architecture Comparison with ECA

In the experiment, three types of well-known networks, namely AlexNet, VGG-16, and ResNet-18, were used as the base frameworks to build and compare the networks. For fairness, the ECA module was added after each convolutional block. Table 4 lists the experimental results, which demonstrate that the accuracy rate obtained when using the VGG-16 architecture to construct the ECACNN is higher than that obtained when using AlexNet and ResNet-18. On this image classification task, the classification accuracy of the ECACNN based on the AlexNet architecture reached 87.56%, while the classification accuracy of the ECACNNs based on the ResNet-18 and VGG-16 architectures obtained 92.32% and 94.04%, respectively. This result emerged that the depth of architectures from AlexNet to VGG-16 was increased, enabling the extraction of high-level and complex abstractions’ information from raw data. It should be noted that ResNet-18 and VGG-16 have a similar size in depth, but ResNet-18 has a substantially lower level of complexity than VGG-16, which is a significant advantage. Specifically, the number of parameters of ResNet-18 (11.18 million) is equivalent to only 13.0% of the parameters in VGG-16 (86.03 million). Therefore, in this study, ResNet-18 was chosen as the backbone of the ECACNN.

### 4.4. Results of Comparing ECADCL with Other Methods

To evaluate the effectiveness of the proposed model, we compared with two traditional models and other classical convolutional neural models for tool wear monitoring based on machined surface images. Traditional methods included principal component analysis (PCA) and genetic algorithm-support vector machine (GA-SVM) traditional handcraft method, GLCM features and GA-SVM traditional handcraft method. Traditional machine learning techniques are constructed by manually extracting features and machine learning models. For feature extracting, the GLCM features extracted as the handcraft methods are specified in Table 5. The PCA method for feature extraction set the accumulative variance percentage at 0.95 as default. At the same time, the machine learning models by using GA-SVM when analyzing extraction features for monitoring the three different levels of cutting tool. The popular deep learning methods of AlexNet, Vgg-16, and ResNet-18 were directly applied for classification. The evaluation results of different methods are presented in Table 6, where it can be seen that the deep learning method outperforms traditional handcraft approaches in classifying tool wear state.

The superiority of the DCL approach for tool wear monitoring is investigated in this section. A second experiment was carried out to determine the precise impact of the proposed model. We designed additional models by using the ECACNN as the backbone network and introducing either the destruction stream or the construction stream individually. The experimental results are shown in Table 6. The accuracy rate of the proposed ECADCL method is 99.96%, representing a significant boost in performance. In our proposed method, destruction learning (DL), which enables discrimination from noisy, detailed, and global features, is verified to help improve the performance of classification. Likewise, the shapes and compositions of the formations of objects modelled by construction learning (CL) can further enhance the performance of the classification model.

### 4.5. Ablation Experiments

In order to prove the feasibility of the proposed model, an ablation experiment is utilized for validation of different parts in ECADCL, including building four modules and comparing their recognition accuracy. The SE module, as channel attention, was compared with ECA in the feature extraction module. The experimental results of each module are recorded in Table 7.

As shown in Table 7, the ablation experiment demonstrates that the performance improvement caused by RCM prove that learning the detailed visual pattern from a “shuffled” visual feature space is beneficial to recognition texture. The ablation experiment also demonstrates that the feature learning ability of the ECA module is better than the SE in the feature extracting module. It can be observed that the ECA’s destruction-construction-based image classification is capable of dealing with tool condition monitoring tasks.

### 4.6. Discussion

**Figure 6:**
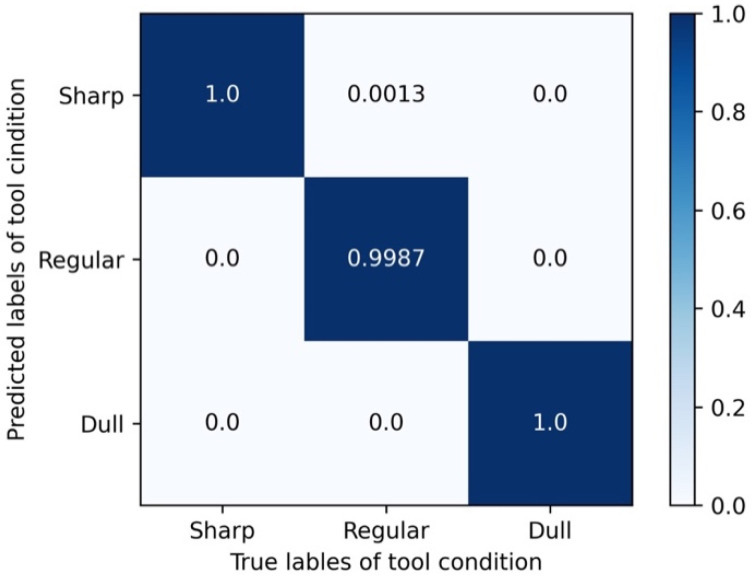
Confusion matrix of the proposed method.

The proposed ECADCL algorithm exhibits excellent performance in tool wear monitoring, and four metrics were used to evaluate the efficacy of the proposed method: confusion matrix, precision, recall, and F-score. The confusion matrix describes a comprehensive overview of the method performance, which is shown in Figure 6. The x- and y-axes reflect the predicted and actual labels, respectively, while the diagonal elements represent the accuracy of each particular state.

**Figure 7:**
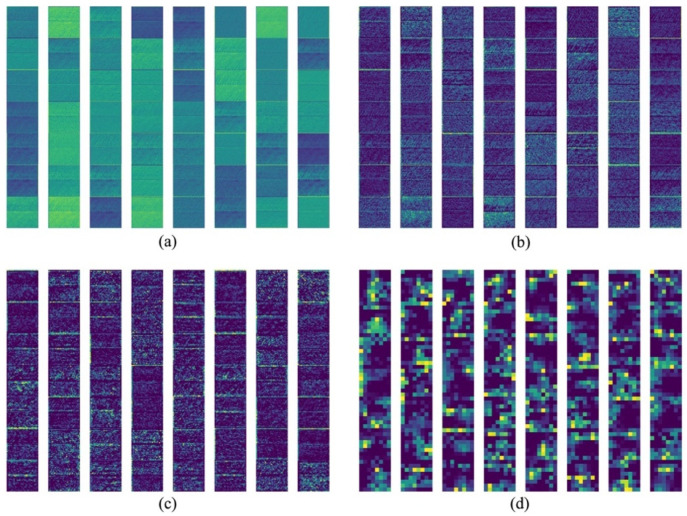
Visualization of the feature maps from (**a**) the last convolutional layer of Conv1_x. (**b**) Last convolutional layer of Conv2_eca. (**c**) Last convolutional layer of Conv3_eca. (**d**) Last convolutional layer of Conv5_eca.

We explore the features learned by ECADCL by visualizing the last convolutional layers of Conv1_x, Conv2_eca, Conv3_eca, and Conv5_eca in Figure 6. In Figure 7a, it can be seen that filters are primarily concerned with extracting texture characteristics, which enables the proposed method to extract more complicated features from deeper layers. From Figure 7b–d, it is noted that more specific information is provided from the superficial layers to the deeper layers.

In addition, we focus on how the running time changes with respect to the model. Deep learning algorithms have the advantage of being super-fast at testing. In order to highlight the superiority of the proposed method in the field of monitoring tool wear, the running time was compared to ResNet-18 and ECACNN on the 5400-sample dataset. The running time of ECADCL, ECACNN, and ResNet-18 are 78 s, 76 s, and 73 s, respectively. Experiments results demonstrated that, compared to other classification algorithms in the control group, the proposed method greatly improved accuracy without losing computation time.

## 5. Conclusions

In this article, we propose a new tool wear monitoring framework with an emphasis on fine-grained categorization and deep learning technology. AlexNet, VGG-16, and ResNet-18, three types of classical deep learning architectures, were researched and compared as a base network to develop an effective network. Introducing the ECA module into the base network enables the network to focus on more representative parts of the input image while suppressing less critical information. The destruction-construction module is also employed to discriminate local features, because of the similar global structures in the surface texture images. In experiments, our proposed model proves more effective than other methods for monitoring the level of tool wear.

However, to train the proposed model for machined surface-based tool wear state monitoring, a deep learning model requires a large amount of training data to achieve outstanding monitoring performance. As a result, our future work will focus on suitable unsupervised models to overcome this challenge. Meanwhile, in the image acquisition process, since the contamination of chips and crude oils generate machined surface pollution, it poses a great analytical challenge for an intelligent monitoring tool wear system. Furthermore, variable factors, such as various tools and cutting parameters, will be explored to further enhance the suggested method’s universality.

## Figures and Tables

**Table 1:** Machining parameters used in the experiments.

Equipment Model	TC500
Spindle speed	6000 rpm
Cutting depth	0.5 mm
Cutting width	2.0 mm
Feed rate	1500 mm/min
Workpiece material	SUS 316 steel
Tool type	ZMCC PML-4E-D6

**Table 2:** Architectural details of the proposed model based on ResNet-18.

Name	Parameters	Output Size
Input layer	Image size: 256 × 256 × 3	256 × 256 × 3
Conv1_x	Filter: [7 × 7, 64], stride: [2 2]	128 × 128 × 6
Conv2_eca	3×3 max pooling, stride: [2 2]	64 × 64 × 64
	Filter: $\left[\begin{array}{cc} 3\times 3, & 64\\ 3\times 3, & 64\\ \mathrm{ECA} & 64 \end{array}\right]\times 2$	
Conv3_eca	Filter:$\left[\begin{array}{cc} 3\times 3, & 64\\ 3\times 3, & 128\\ \mathrm{ECA} & 128 \end{array}\right]\times 2$	32 × 32 × 128
Conv4_eca	Filter:$\left[\begin{array}{cc} 3\times 3, & 128\\ 3\times 3, & 256\\ \mathrm{ECA} & 256 \end{array}\right]\times 2$	16 × 16 × 256
Conv5_eca	Filter:$\left[\begin{array}{cc} 3\times 3, & 256\\ 3\times 3, & 256\\ \mathrm{ECA} & 512 \end{array}\right]\times 2$	8 × 8 × 512

**Table 3:** The distribution of the dataset.

Dataset	Training Set	Test Set
Sharp	4200	1800
Normal	4200	1800
Dull	4200	1800

**Table 4:** The recognition accuracy of ECACNN based on different architectures.

Network Architecture	Accuracy (%)	Parameters (Million)
AlexNet	87.56	33.64
VGG-16	94.09	86.03
ResNet-18	92.32	11.18

**Table 5:** The handcrafted GLCM features list.

Feature	Formula
Contrast	$\sum_{i,j=0}^{\mathrm{levels}-1}P_{i,j}{\left(i-j\right)}^{2}$
Dissimilarity	$\sum_{i,j=0}^{\mathrm{levels}-1}P_{i,j}\left|i-j\right|$
Homogeneity	$\sum_{i,j=0}^{\mathrm{levels}-1}\frac{P_{i,j}}{1+{\left(i-j\right)}^{2}}$
ASM	$\sum_{i,j=0}^{\mathrm{levels}-1}P_{i,j}^{2}$
Correlation	$\sum_{i,j=0}^{\mathrm{levels}-1}P_{i,j}\left[\frac{\left(i-\mu_{i}\right)\left(j-\mu_{j}\right)}{\sigma_{i}\sigma_{j}}\right]$

**Table 6:** Performance comparison.

Method		Accuracy (%)
Traditional	PCA + GA-SVM	59.56
	GLCM features + GA-SVM	79.80
Deep learning	AlexNet	88.68
	Vgg-16	86.68
	ResNet-18	90.29
	ECACNN	92.32
	Ours, ECADCL	99.96

**Table 7:** The ablation experimental results.

Method	Accuracy (%)
ResNet-18 (baseline)	90.29
+SE	91.61
+ECA	92.32
+ECA+RCM	99.36
+ECA+RCM+ Adversarial Learning (DL)	99.91
+ECA+RCM+ Construction Learning (CL)	99.78
Ours, ECADCL	99.96

## Data Availability

Not applicable.
